# Early Discharge Planning and Improved Care Transitions: Pre-Admission Assessment for Readmission Risk in an Elective Orthopedic and Cardiovascular Surgical Population

**DOI:** 10.5334/ijic.2260

**Published:** 2016-05-24

**Authors:** Brenda Ohta, Ana Mola, Peri Rosenfeld, Shauna Ford

**Affiliations:** NYU Langone Medical Center, USA

**Keywords:** care transitions, elective surgery, discharge planning, readmission risk, care continuum

## Abstract

**Background/Methods:**

Readmission prevention is a marker of patient care quality and requires comprehensive, early discharge planning for safe hospital transitions. Effectively performed, this process supports patient satisfaction, efficient resource utilization, and care integration. This study developed/tested the utility of a predictive early discharge risk assessment with 366 elective orthopedic/cardiovascular surgery patients. Quality improvement cycles were undertaken for the design and to inform analytic plan. An 8-item questionnaire, which includes patient self-reported health, was integrated into care managers’ telephonic pre-admission assessments during a 12-month period.

**Results:**

Regression models found the questionnaire to be predictive of readmission (p ≤ .005; R^2^ = .334) and length-of-stay (p ≤ .001; R^2^ = .314). Independent variables of “lives-alone” and “self-rated health” were statistically significant for increased readmission odds, as was “self-rated health” for increased length-of-stay. Quality measures, patient experience and increased rates of discharges-to-home further supported the benefit of embedding these questions into the pro-active planning process.

**Conclusion:**

The pilot discharge risk assessment was predictive of readmission risk and length-of-stay for elective orthopedic/cardiovascular patients. Given the usability of the questionnaire in *advance* of elective admissions, it can facilitate pro-active discharge planning essential for producing quality outcomes and addressing new reimbursement methodologies for continuum-based episodes of care.

## Purpose

Whereas a surgical hospitalization can be categorized as a discrete medical episode, there are crucial pre-hospitalization, inpatient, and post-hospitalization transition points that require integration and communication across care settings and clinical providers. These may serve to influence the overall quality and safety of patient care and rehabilitative outcomes, including readmission. This pilot quality improvement study was conducted to facilitate an improved process for patient risk assessment and early discharge planning in order to: 1) inform and integrate care across the pre-, inpatient, and post-hospital settings 2) improve patient experience and outcomes, and 3) engage patients/their caregivers in the patient care planning process. Specifically, our pilot developed and tested the utility of an early discharge risk assessment questionnaire applicable to pro-active pre-admission discharge planning for elective orthopedic/cardiovascular surgery patients. Furthermore, in addition to its potential for improving quality patient outcomes, the pro-active approach to discharge planning piloted in this study is immediately relevant to new reimbursement models emerging in the U.S. Medicare market that require enhanced coordination and efficiency across the continuum of care^1^ [1].

## Background

Prevention of hospital readmission is increasingly viewed as a marker of patient care quality, and identified by health plans as a key component of value-based reimbursement methodology, including new models for U.S. Medicare reimbursement under a comprehensive care payment model^1^ [1,2,3,4,5]. The discharge planning process, when conducted in a timely manner and with a focus toward patient/family engagement, supports integrated care, efficient patient flow across care transitions, appropriate/cost-effective use of resources, and optimizes both reimbursement and clinical quality [3,6,7,8,9,10,11,12,13]. To effectively plan for a safe, timely, and appropriate hospital transition, it is essential to accurately identify factors that predict and contribute to patient risk for extended hospitalization and readmission.

Unfortunately, currently available risk assessment models and tools are not readily applicable to a general medical/surgical population [14,15,16,17,18]. Some lack flexibility or generalizability across hospital entry points/timeframes and patient populations [17,19,20,21,22,23]. For example, some screening tools demonstrating efficacy in predicting 30-day readmission risk for general medical populations are not applied until at/near hospital discharge, thereby precluding opportunities for early discharge planning for non-elective patients, or pre-admission planning for elective patients at highest risk [21,22]. In addition, studies of assessment tools developed for the pre-hospital/emergency department settings may not examine readmission risk, nor be applicable to the inpatient setting [20,23,24]. Other tools/methods have limited use in the clinical setting due to complexity in scoring or need for retrospective data (i.e., a completed hospital length-of-stay, coded clinical data available after discharge, follow-up with the primary care doctor after discharge, etc.) [17,21]. Lastly, effective readmission risk tools can be found, but for specific populations, such as the LACE for congestive heart failure, leaving a gap in application to other selected patient populations or the patient population in general [6,9,15,16,24].

One meta-analysis, in particular, identified 26 unique models for readmission risk assessment [17]. Many models were found to have poor discriminative ability, some were designed for use at hospital admission only, and others relied on retrospective administrative data or were intended for use only upon discharge which does not provide adequate time for safe and timely discharge planning. The results of the meta-analysis suggest that the majority of the readmission risk models, focusing primarily on chronically ill medical patients, performed poorly. Furthermore, while some research can be found that includes patient self-report of health variables in relation to readmission risk, existing tools generally neglected to include patient self-assessment of health which may in fact be a crucial indicator of readmission risk and other important outcomes [17,20,21,26].

Undoubtedly, continued work to improve performance in this area is required to develop better, not only more comprehensive approaches to risk assessment, but to improve the discharge planning process as well. In fact, Allaudeen et al (2011) discuss the need for a systematic and consistent discharge process using risk factor data for targeting quality improving efforts [20]. These are especially needed for hospital professionals such as nurse and social work case/care managers who provide care coordination, transitional care and other clinical services that contribute to timely, safe discharge planning and prevention of re-hospitalization. The absence of standardized processes/tools for pre-admission/early hospitalization use, and for the elective surgical patient population, is especially notable upon review of the literature. Given increasingly short hospital stays, limited time for patient engagement and safe discharge planning, and changing reimbursement models which “bundle” hospital payments to include the pre-, inpatient, and up to 90 day post-hospital care services related to a single episode of care (such as an elective hip replacement surgery), the need to develop tools and processes for discharge efficiency and safety is essential. Improved tools and processes to enhance discharge planning and identify readmission risk can assist busy clinicians in focusing efforts on those patients with the greatest needs, thereby contributing to improved patient outcomes and experiences, including elective surgical patients who often have very brief hospital stays and would otherwise have had insufficient time to plan and prepare. This study, therefore, focused on the development of a brief discharge planning risk assessment questionnaire, embedded into an early pre-admission screening process, for use prior to an elective surgical hospitalization.

## Study Design and Methods

### Setting and Study Population

This quality improvement pilot was conducted by the Care Management Department at NYU Langone Medical Center (NYULMC), a major academic medical center in New York City. NYULMC is a 1000+ bed multi-hospital system that provides tertiary and quaternary services to a diverse urban population. The patient target population for this initiative was 366 adult patients, aged 25 and above, electing to have orthopedic hip or knee replacement surgery or cardiac valve surgery. These are high-volume surgical populations for our medical center.

Excluded from this study were pediatric patients and non-elective emergent/urgent patients. No patients were eliminated from this study based on their performance on the risk questionnaire; the entire pool of available, electively admitted surgical hip and knee patients were therefore included in this evaluation.

With guidance from the NYU School of Medicine IRB, this initiative falls within the domain of quality improvement and not considered human subjects research. It was not a randomized, controlled trial. Thus, the project did not require IRB review and meets our institution’s standards for ethical adherence in research.

### Research Questions

The study was designed to answer the following specific research questions: 1) Can a brief questionnaire be developed to identify early readmission risk factors for more effective discharge planning; 2) Is there a statistically significant relationship between patient’s self-rating of health and readmission risk for elective surgical patients; and 3) Can these questions be used to identify risk for readmission and increased hospital stay prior to admission or early in the hospital stay?

### Design phase/Pre-implementation

A continuous quality process improvement approach^2^ was employed by the Care Management (CM) team to develop, test, redesign, retest and finalize a discharge risk assessment questionnaire that would be embedded into a pre-admission assessment and early discharge planning process for a population of patients prior to elective surgical hospital admission [26]. Items from patient questionnaire and risk assessment tools from the literature were reviewed and evaluated for use by the CM team for this pilot producing a final questionnaire based on standardized, objective data elements [19,21,23,26,28,29,30,31,32,33,34,35,36,37]. The charge of the CM team was not to develop an entirely new risk tool for the purposes of producing a cumulative risk score. Rather, it was to develop a brief questionnaire based on previously published and clinically accepted patient variables and questions that are likely to be associated with readmission and utilization risk and would thereby support the process for early discharge planning for elective surgical patients. The resulting set of items (refer to Table 1) identified by the CM team as being crucial to this charge addressed: “lives alone,” “pain,” “prior hospitalization,” “depression,” “functional status,” “high risk medications,” and “health literacy.” A final item regarding patient perception of his or her overall health was also included in order to examine the predictability of patient self-reported well-being on discharge outcomes [21,26,28].

**Table 1:** Assessment of Factors Associated with Readmission Risk.

Questions*	

1.	Do you live alone [23]? (*Yes, no*)
2.	Self-rated health: In general, would you say your health is: excellent, very good, good, fair, or poor [26,28]?
3.	Right now, on a scale of 0 to 10, with 0 representing no pain, 5 moderate pain, and 10 the worst pain imaginable, how much pain do you have [29]?
4.	Have you had a hospitalization or emergency dept visit in the last year [19,21]? (*Yes, no*)
5.	Over the last 2 weeks, how often have you felt bothered by any of the following: a) Little interest or pleasure in doing things; b) Feeling down, depressed, or hopeless [30]? (*scale: 0 to 3; not at all to nearly every day*)
6.	Functional Status: a)Can you get out of bed or chair yourself; b) can you dress and bathe yourself; c) can you make your own meals: d) can you do your own shopping [31,32]? (*Yes, no*)
7.	Are you taking any of the following medications? Pills that impact your blood clotting (Coumadin, aspirin, Plavix), Insulin/blood sugar pills, or prescription pain meds [33,34]? (*Yes, no*)
8.	Health literacy: How often do you need to have someone help you when you read instructions, pamphlets, or other written material from you doctor or pharmacy [35,36]? (*Never, rarely, sometimes, often, always*)

*For discharge planning, the following responses were used to indicate potential for patient risk: a) For questions 1, 4, 6, 7, a “yes” response for any item. b) For question 2, a response of “fair” or “poor.” c) For question 3, a score of 5 or higher. d) For question 5, a score of 1 or higher for either or both questions. e) For question 8, a response of “sometimes”, “often” or “always.”

## Study Design and Implementation

The eight risk assessment questions were integrated into the care manager pre-admission discharge planning assessment process which was conducted telephonically for patients one-to-four weeks prior to elective admission for orthopedic and cardiac surgeries scheduled during 2013–2014. Names of patients assigned to the surgery pre-admission clinic registration for hip, knee, or cardiac valve replacement were forwarded to the nurse care managers responsible for these patient groups. Patients were contacted by the care managers within 72 hours of the patient name being added to the schedule. This could occur anytime between one-to-four weeks prior to surgery as determined by the surgeon in collaboration with the patient. Care managers contacted patients via phone to conduct the screening assessment, discuss what to expect during hospitalization, review methods for active patient/family participation and how to prepare for surgery, establish expectations for hospital length-of-stay, and prepare a mutually agreed upon plan for care goals and for discharge from the hospital.

## Evaluation/Analysis

Readmission and hospital length-of-stay outcomes were retrospectively evaluated in relationship to the patient discharge risk questionnaire responses. Regression analyses were performed using SPSS version 23 to analyze data and an alpha level of .05 was set for all statistical tests. Stepwise logistic regression analysis was performed to determine which independent variable predicted likelihood of readmission within 30 days of discharge. Multivariate regression analysis was performed to examine predictive power of the questions vis-à-vis hospital utilization (proxy utilization measure = length of stay). Transformation of the length of stay (LOS) dependent variable was required for the regression analysis; LN (LOS) was used to adjust for the non-normal distribution in hospital length of stay data in order to conform to the assumptions of the regression model. For purposes of results discussion in this paper, LOS refers to the transformed variable of LN (LOS).

Stepwise models examined the following independent variables: demographic variables (patient age, gender, race/ethnicity) and health insurance type; clinical variable (orthopedic or cardiac surgery); plus social/functional variables (lives alone, self-rated health, prior hospitalization, functional status, high risk medications, depression, pain, and health literacy).

Quality process measures were also retrospectively evaluated for (i) patient experience with the transition of care and discharge as reported through the Hospital Consumer Assessment of Healthcare Providers and Systems (HCAHPS) patient experience survey^3^ [38]; the Pearson chi-square test was used to determine if there was a statistically significant association between pre- and post- pilot patient experience scores, (ii) the efficacy of the discharge process (i.e., care manager accuracy in predicting best option for patient discharge pre-admission compared to patient’s actual discharge disposition, and (iii) discharge disposition type (the percentage of patient’s able to be discharged directly home compared to an interim skilled nursing or rehabilitation facility). The measure of patient experience addresses the potential impact on the patient related to changes in the process of early discharge planning. The process measures, items (ii) and (iii), address the need for reduced variation and greater efficiency in the new processes developed through the quality improvement effort. These measures were evaluated in the period prior to the start of the pilot study and after the conclusion of the pilot study, specifically for the orthopedic surgical patients. Process measures data for cardiovascular surgery patients were not available prior to the start of the pilot study, precluding us from making similar comparisons.

## Results/Findings

### Sample Description

The majority of patients (89%) were elective orthopedic; 11% were elective cardiac. Payers included Medicare (41% Medicare fee-for-service; 12% Medicare Advantage), Medicaid (5%), and commercial plans (42%). A majority (59%) of patients were female with 41% of the patients being male. 44% of the patients were age 65 and older with the mean age being 65, median and modal age being 66. The average hospital length of stay for the study patients was 3.59 days (cardiac = 6.61 days; orthopedic = 3.26 days). The overall 30-day readmission rate for all patients in the pilot was 3.8% (cardiac = 12.5%; orthopedic = 2.8%).

### Stepwise Logistic Regression: Predicting Readmission

The stepwise logistic regression model (Table 2) was predictive of readmission (p < .005, R^2^ = .334) and explained 33.4 percent of the variance in the outcome measure (i.e. readmission among study population). While cardiac patients, as expected due to their clinical complexity, had over 11.0 times greater odds of being readmitted, the analysis (Model Five) demonstrated that when controlling for this and all other variables in the equation, patients who live alone have nearly a 5.0 times greater odds of readmission (p ≤ .05) and patients who have poor/fair self-rated health have approximately an 18.0 times greater odds of readmission (p ≤ .001).

**Table 2:** Logistic Regression Analysis: Odds Ratios of Readmission.

Independent Variables	Model 1 OR (CI)	Model 2 OR (CI)	Model 3 OR (CI)	Model 4 OR (CI)	Model 5 OR (CI)

**Age**	1.02 (.97–1.01)	1.00 (.94–1.07)	.99 (.93–1.05)	.98 (.91–1.04)	.97 (.91–1.04)
**Gender (ref. male)**					
Female	1.14 (.36–3.63)	1.10 (.35–3.53)	1.52 (.44–5.22)	.78 (.20–3.12)	.87 (.21–3.68)
**Race/Ethnicity (ref. white)**					
Black	1.28 (.25–6.48)	1.33 (.26–6.75)	2.04 (.36–11.52)	2.45 (.36–16.92)	1.84 (.22–15.27)
Asian	3.57 (.38–33.06)	3.75 (.40–35.11)	3.78 (.36–39.60)	2.97 (.17–52.75)	3.31 (.12–88.01)
Other Race/Ethnicity	.99 (.20–4.82)	1.04	1.57 (.29–8.54)	.90 (.15–5.54)	1.15 (.16–8.09)
**Insurance** (ref. Medicare FFS)					
Other		.59 (.15–2.33)	.91 (.20–4.26)	.79 (.15–4.32)	.72 (.13–4.12)
**Surgery** (ref. Orthopedic)					
Cardiac			7.66** (1.66–35.40)	14.83*** (2.45–89.78)	11.71* (1.64–83.50)
**Lives Alone^**				5.12* (1.40–18.69)	4.96* (1.31–18.72)
**Self-Rated Health^**				14.65**** (3.46–62.00)	18.24**** (3.39–98.20)
**Pain^**					.59 (.12–2.87)
**Prior Hospitalization** (last 12 months)^					1.59 (.39–6.58)
**Depression^**					.58 (.05–6.29)
**Functional Status^**					1.58 (.30–8.32)
**High Risk Medications^**					.55 (.12–2.49)
**Health Literacy^**					.41 (.52–3.17)
Constant	.01***	.04	.03	.02	.04
**df**	5	6	7	9	15
**Nagelkerke R²**	.02	.02	.09	.31****	.33***
**n = 366**					

*p ≤ .05; **p ≤ .01; ***p ≤ .005; ****p ≤ .001. ^ Items comprising discharge risk screening tool. Odds ratio, Exp(B), is a relative measure of effect of an outcome occurring given a particular exposure, compared to the odds of the outcome occurring in the absence of that exposure.

Model Four, with its statistically significant contributions for “lives alone” and “self-rated health” (two out of eight risk screening questions) accounts for over 30% of the variance. Model Five incorporates the remaining 6 out of 8 risk screening questions. As can be seen from the results in Table 2, while statistically significant, these add only minimal improvement in model fit (refer to Table 2, Models Four and Five).

### Stepwise Multiple Regression: Predicting Length of Stay

The multiple regression model (Table 3) was predictive of increased length of stay (p ≤ .001; R^2^ = .314) and revealed that poor/fair self-rated health was a significant predictor of increased length of stay (B = .137; p = .01). The stepwise regression model explains 31.4 percent of the variance in length of stay. The independent variable of self-rated health was highly predictive of increased length of stay (p ≤ .05).

**Table 3:** Multivariate Regression Analysis: Hospital Length of Stay.

Independent Variables	Model 1 β	Model 2 β	Model 3 β	Model 4 β	Model 5 β

**Age**	.01****	.00	.00	.00	.00
**Gender** (ref. male)					
Female	–.07	–.08	–.30	–.04	–.04
**Race/Ethnicity** (ref. white)					
Black Asian OtherRace/Ethnicity	.02 .10 .04	.03 .11 .05	.09 .06 .11*	.12 .04 .08	.12 .03 .07
**Insurance** (ref. Medicare FFS)		–1.28*	–.02	–.03	–.04
Other					
**Surgery** (ref. Orthopedic)			.68****	.68****	.67*
Cardiac					
**Lives Alone^**				–.04	–.04
**Self-Rated Health^**				.14**	.13*
**Pain^**					–.01
**Prior Hospitalization** (last 12 months)^					.02
**Depression^**					.03
**Functional Status^**					–.01
**High Risk Medications^**					–.03
**Health Literacy^**					.05
Constant	.75****	1.03****	.95****	.96****	.98****
**df**	5	6	7	9	15
**R²**	.05**	.07****	.29****	.31****	.31****
**n = 366**					

*p ≤ .05; **p ≤ .01; ***p ≤ .005; ****p ≤ .001. ^ Items comprising discharge risk screening tool. Length of stay measured in days. Beta coefficients (β) refer to how much the dependent variable (ie., length of stay) will change per standard deviation increase or decrease in the independent, or predictor, variable.

### Related Patient Quality Measures: Patient Experience and Discharge Disposition

Other measures of effectiveness included (i) patient experience with care transitions and discharge, (ii) care manager accuracy in predicting best option for patient discharge pre-admission compared to patient’s actual discharge disposition, (iii) the percentage of patient’s able to be discharged directly home compared to an interim skilled nursing or rehabilitation facility. Specific findings related to these measures are: Examples of these associated measures of program effectiveness exhibited positive results as follows:

- Elective orthopedic hip and knee surgery patient experience scores for satisfaction with the care transition process increased from the 50^th^ percentile to the 60^th^ percentile for an overall increase of 20% between the time of the start of the pilot in 2013 compared to the experience scores received after the conclusion of the pilot in 2014 (p > .05 at p = .068). For the same time period, patient satisfaction with discharge increased from the 88^th^ percentile to the 95^th^ percentile, for an overall increase of greater than 7% (p < .05 at p = .013).
- Percentage of knee replacement patients being discharged directly to home, rather than to skilled nursing or acute rehabilitation care, increased from 42% (pre-pilot) to 60% after the conclusion of the pilot.
- Percentage of pre-admission discharge plans that matched the actual discharge disposition increased from 62% (pre-pilot) to 82% after the conclusion of the pilot for knee replacement patients.

### Study Limitations and Next Steps

This study of a discharge risk questionnaire and early planning process was piloted on a specific orthopedic and cardiac surgery population. The generalizability of its findings to the broader patient population is therefore limited. In addition, although our study examined a sample of 366 patients, the low rate of readmission occurrences, while certainly desirable from a clinical perspective, necessitates caution in interpreting these findings. The discriminatory power of the discharge risk questionnaire, tested on a population with a relatively low number of outcome events (i.e., readmission rates), could not be examined. Further application of the risk questionnaire to a larger and more diverse sample would enhance generalizability and allow for more advanced analysis of the psychometric (e.g., test-retest) and analytic (e.g., discriminatory) properties of the questionnaire.

With regard to a potential source of confound, it should be acknowledged that the questionnaire’s implementation could itself have influenced the outcome it was aiming to predict. In this way, the questionnaire’s administration at pre-admission may have contributed to the patient’s course of treatment, discharge, and readmission. Indeed, this should have been expected and desired given that the primary goal of the clinical staff was to optimize patient care. This systematic bias, however, would have served to weaken, not strengthen, observed relationships between questionnaire responses and outcome measures because clinical intervention would have compensated for any anticipated need of the patient. Consequently, any relationship that emerged as statistically significant can be viewed as especially robust given that the direction of contamination bias would have “stacked the deck” against it.

As mentioned, future studies with a larger and more diverse patient population will be required to further elucidate the utility of this questionnaire. Given the timeframe and scope of this pilot quality improvement study, however, its statistically significant findings and its pre-admission discharge process nonetheless provide a useful starting point for care transitions redesign efforts for hospitals.

## Discussion

Three primary lessons can be gleaned from this pilot study: 1) Early evaluation of patient utilization and readmission risk is possible and has the potential to impact the quality and safety, and cost of patient care transitions: 2) Engagement of the clinician (i.e., the nurse care manager) is required for a comprehensive, effective workflow to improve patient care planning and transition management; and 3) Direct patient input regarding self-rated health is a key variable in assessing patient risk for extended utilization and readmission risk. Each of these three points is discussed in more detail below.

To address the first point, in this pilot study, the discharge risk assessment questionnaire was administered in advance of hospital admission. Such pre-admission discharge risk assessment is a novel approach allowing clinicians to engage patients and their caregivers at the earliest possible point in the care trajectory for targeted discharge planning. This is highly important in order to quickly identify and help to mitigate factors which may place surgical patients at risk for adverse outcomes including re-admissions. Results demonstrated that the questionnaire holds promise in predicting elective surgical patients with higher hospital utilization and readmission risk. The benefits of early assessment are evident for both the care manager and patient in allowing them to collaborate in planning for more patient-centered discharges and aligning expectations for care planning and goal setting, which may positively impact patient satisfaction, safety, and overall experience.

While a risk assessment questionnaire that can be initiated at the patient’s earliest point of entry into the healthcare system may positively improve discharge and care transition planning, it single-handedly, will not transform the quality of care transitions nor reduce readmissions. Nor can it be developed in isolation of the clinicians who carry out this essential work. This is the second important lesson of our pilot study: the need to centrally involve the clinical nurse care manager in the development process for both the risk questionnaire and the associated discharge planning workflow. The partnership of nurse care managers was of vital importance to this pilot initiative. The final risk questionnaire and discharge planning model was clinically driven by our team of care managers, producing a questionnaire and approach that increased care manager ownership in, and follow-through with, the process. As reported by the care managers, the availability of an easy-to-use discharge risk screening questionnaire was an effective method to facilitate patient engagement, initiate further discussion related to patient goals and preferences, and could be applied in real time to prepare the patient in developing an appropriate plan for safe discharge and care transition.

Given that patients do not experience their care needs as discrete episodes (i.e., the surgical experience may not in fact be viewed separately from the quality of recovery experienced after discharge), a comprehensive care management/transitional care effort is required which employs skilled clinical care managers to conduct effective preadmission planning, careful communication and coordination throughout the inpatient stay, and diligent discharge planning follow-through, inter-agency/provider interface, and patient outreach in the immediate post-acute care days. Inclusion of clinicians, from the very start of the process, is critical to overall success of the initiative and ultimately to the successful transition of patients across the healthcare continuum.

Thirdly, and possibly the most important finding, the study demonstrates that directly soliciting the patient’s rating of his/her health is a key predictor associated with risk of re-hospitalization and utilization of hospital resources. While clinical, utilization, and demographic variables are common to high risk assessment, patient self-reported variables are not as common. The implication is clear: Patient input is important in assessing risk and in the discharge planning process. This pilot demonstrated that patients can be reliable sources of information about their own health and their self-assessments can help to predict outcomes. In our study, patient self-assessment of health and living alone were statistically significant risk factors that may be considered “early warning signals” of those patients with highest risk of readmissions or longer hospital utilization. Other variables, such as depression, functional status, previous admission history, etc., while expected to yield statistically relevant findings based on previous results documented in the literature, did not. This may be due to the mitigating effect of the care manager’s role in pre-admission planning and the level of patient engagement. It may also be related to sample size, or lack of generalizability of previous research findings to our specific surgical population. These non-statistically significant variables, however, did contribute to the overall positive findings of our model, and remain useful in providing information necessary for effective discharge planning and overall care coordination/care transition processes.

## Conclusion

This unique pre-admission risk questionnaire was found to be predictive of readmission risk and increased length of stay in a pilot quality improvement study with two elective surgical populations. Embedded into an early discharge planning process which incorporated mutually agreed upon patient-clinical goals, related findings also support the questionnaire’s utility in improving patient satisfaction with discharge and improvements in the percentage of patients able to be discharged directly home, rather than discharged to nursing facilities following surgery.

Given the flexibility of this questionnaire to be administered in advance of an elective surgery admission, it has the potential to support pro-active discharge planning decision making and early intervention to address patient needs in advance of hospitalization and surgery. Such efforts are relevant not only to improved care quality, but to delivery of effective, integrated care transitions critical under new healthcare delivery and reimbursement models.

## Footnotes

Comprehensive Care Payment Model: U.S. Centers for Medicare & Medicaid Services (CMS) implemented the Comprehensive Care for Joint Replacement payment model on April 1, 2016, beginning with Joint Replacement surgery. This new reimbursement method “bundles” the payment for the episode of care related to the joint replacement surgery from the date of surgery until 90 days following hospital discharge. Joint Replacements are the first of what is expected to be a number of procedures and diagnoses CMS will convert to this new payment model over time. Hospital performance related to clinical quality measures, discharge disposition/cost of post-hospital care, readmissions, and other clinical or utilization outcomes can potentially impact reimbursement under this methodology. Care redesign related to patient risk identification, discharge planning, care transitions, and continuum-based services is a key factor for readiness for this new payment methodology [1].

Continuous Quality Process Improvement Cycle: Our study utilized the FOCUS-PDCA approach, an acronym which represents The Deming Cycle or Shewhart Cycle. This model of continuous quality improvement begins with the FOCUS stage: Find an opportunity to improve; Organize a team; Clarify current knowledge and process; Understand variation in process, and Start PDCA/PDSA cycles to improve. PDCA/PDSA refers to: Plan or pilot test the new action/intervention; Do, or implement, the action/intervention; Check, or Study, the outcomes by collecting and analyzing data related to the intervention; and Act by implementing the intervention or process changes if the analysis finds them to be effective, or repeat the PDCA cycle for further improvements/refinement [27].

HCAHPS/Press-Ganey Patient Experience Survey: Hospital Consumer Assessment of Healthcare Providers and Systems (HCAHPS) patient experience survey, available through authorized vendors such as Press-Ganey Associates, Inc., is a part of the value-based purchasing initiative required under the U.S. Affordable Care Act/2010 national health care reform legislation. It allows healthcare consumers to provide feedback on their experiences of care across key domains such as care from doctors and nurses, environment of care, discharge and care transitions, as well as overall ratings. Scores can be reported as percentile ranks which reflect hospital performance in relation to its peer hospitals. Movement upward in percentile ranking generally require substantial organizational improvement efforts [38].
